# Anion-Controlled Architecture and Photochromism of Naphthalene Diimide-Based Coordination Polymers

**DOI:** 10.3390/polym10020165

**Published:** 2018-02-08

**Authors:** Jian-Jun Liu, Shu-Biao Xia, Yu-Lian Duan, Teng Liu, Fei-Xiang Cheng, Cheng-Ke Sun

**Affiliations:** Center for Yunnan-Guizhou Plateau Chemical Functional Materials and Pollution Control, Qujing Normal University, Qujing 655011, China; xiashubiao401@163.com (S.-B.X); dyl_0226@126.com (Y.-L.D); 15288404381@163.com (T.L.); chengfx2010@163.com (F.-X.C.); cksun99@126.com (C.-K.S.)

**Keywords:** coordination polymer, anion, naphthalene diimide, photochromism, electron transfer

## Abstract

Three new cadmium coordination polymers, namely [Cd(NO_3_)_2_(DPNDI)(CH_3_OH)]·CH_3_OH (**1**), [Cd(SCN)_2_(DPNDI)] (**2**), and [Cd(DPNDI)_2_(DMF)_2_]·2ClO_4_ (**3**) (DPNDI = *N*,*N*-di(4-pyridyl)-1,4,5,8-naphthalene diimide, DMF = *N*,*N*-dimethylformamide) have been synthesized by reactions of DPNDI with Cd(NO_3_)_2_, Cd(SCN)_2_, and Cd(ClO_4_)_2_, respectively. Compound **1** is a one-dimensional coordination polymer with strong lone pair-π interactions between the coordinated NO_3_^−^ anions and the imide ring of DPNDI; while **2** is a two-dimensional network with a (4, 4) net topology. In the case of **3**, due to the presence of uncoordinated perchlorate counter ions, it exhibits a non-interpenetrated square-grid coordination polymer containing one-dimensional rhomboid channels. The structural diversity in these compounds is attributed to different coordination abilities and geometries of counter anions. Due to the presence of electron-deficient NDI moiety, the photochromic behavior of these compounds was studied. Interestingly, only compounds **1** and **3** exhibit color changes under light irradiation. The influence of the anions on the photochromism process of the NDI-based materials has been discussed.

## 1. Introduction

Due to the versatility of coordination polymers in crystal structures and chemical compositions, they have developed into an important class of materials and enable a wide range of applied properties, such as catalysis, guest/ion-exchange, fluorescence, adsorption, and separation [1,2,3,4,5,6,7,8]. So far, although a large number of coordination networks have been reported, synthesis of coordination polymers with desired topology still remains a long-term challenge to chemists because many factors, such as pH value, solvents, temperature, and counter ions affect the final structures of the coordination polymers [9,10,11,12,13,14]. As we know, anions play important roles in the formation of coordination polymers. For example, some anions, such as X^−^, NO_3_^−^, and CH_3_COO^−^ with strong coordination ability can coordinate to metal ions or bridge metal centers to generate multi-nuclear units or high-dimensional frameworks [15,16,17,18], but some other anions, such as PF_6_^−^, ClO_4_^−^, BF_4_^−^, and CF_3_SO_3_^−^ possess very weak coordination ability and usually act as counterions and templates in the formation of the products [19,20,21]. Although many studies on anion-controlled formation of coordination polymers have been reported, very few examples of anion effect on the photochromic properties of coordination polymers have been involved so far because it is usually difficult to create appropriate coordination polymers with photochemical activity, but also containing different anions [22,23,24].

Naphthalene diimide (NDI) possess π-conjugated planes, high redox activity, and strong π-acidity, which are excellent candidates of organic ligands for the construction of photochromic coordination polymers due to the controllability of the substituent groups on the diimide nitrogens and the reversibility of the electron transfer [25,26,27]. Many groups have been used as NDI derivatives as catalysts through anion-π catalysis, and as organic ligands to construct coordination polymers [26,28,29,30,31,32,33]. In order to investigate the detailed effect of different anions on the structures and photochromic properties, and as coordination polymers constructed by the neutral ligands have close relationship with the counter anions [34], we chose a neutral ligand *N,N*-di(4-pyridyl)-1,4,5,8-naphthalene diimide (DPNDI) as an organic ligand. The counter anions can greatly influence the resultant supramolecular architectures because they can either coordinate to the metal center or accommodate in the frameworks for charge-balance requirements. As expected, three Cd(II) coordination polymers, namely [Cd(NO_3_)_2_(DPNDI)(CH_3_OH)]·CH_3_OH (**1**), [Cd(SCN)_2_(DPNDI)] (**2**), and [Cd(DPNDI)_2_(DMF)_2_]·2ClO_4_ (**3**) were obtained when reaction of DPNDI with Cd(NO_3_)_2_, Cd(SCN)_2_, and Cd(ClO_4_)_2_, respectively, in which **1** is a one-dimensional linear network showing strong lone pair-π interactions, **2** is a two-dimensional networks with the (4, 4) net topology, while **3** is also a two-dimensional network showing a one-dimensional rhomboid channel. It was found that counter anions significantly affect the final structureof the crystal lattice. Moreover, the influence of the anions on the photochromism process of these compounds has been also discussed.

## 2. Experimental Section

### 2.1. Materials and Methods 

All chemicals and reagents were used as received unless otherwise stated. The organic ligand DPNDI was synthesized according to the same procedures reported in the literature [35]. The infrared spectra were obtained in the range of 400–4000 cm^‒1^ from a Perkin-Elmer FT-IR spectrophotometer (Waltham, MA, USA), and powder X-ray diffraction (PXRD) were recorded on a Rigaku MiniFlex-II X-Ray diffractometer (Tokyo, Japan) using graphite-monochromated Cu Kα radiation (λ = 1.5406 Å) in the range of 5–50°. TGA measurements were performed on a TG-209 system (DuPont, Wilmington, DE, USA), with a heating rate of 10 °C/min with N_2_ atmosphere. The electron spin resonance (ESR) measurements were obtained on a Bruker A300 instrument (Billerica, MA, USA) operating in the X-band at room temperature using powder crystal material. Luminescent properties were recorded on an Edinburgh Instrument FLS 920 luminescence spectrometer (Livingston, UK). UV–Vis diffuse reflectance spectra were recorded at room temperature on a Perkin–Elmer Lambda 900 UV–Vis spectrophotometer (Waltham, MA, USA) equipped with an integrating sphere by using BaSO_4_ as a white standard in the range of 300–800 nm. 

### 2.2. Synthesis of [Cd(NO_3_)_2_(DPNDI)(CH_3_OH)]·CH_3_OH *(**1**)*

A solution of Cd(NO_3_)_2_·4H_2_O (61.6 mg, 0.2 mmol) in CH_3_OH (5 mL) was carefully layered onto a solution of DPNDI (21.0 mg, 0.05 mmol) in *N,N′*-dimethylacetamide (5 mL) in a test tube. The solution was left to stand for several days in the dark at room temperature, and light-yellow crystals of **1** were obtained (yield: 42% based on DPNDI). Anal. Calcd for C_28_H_20_CdN_6_O_14_: C 43.25, H 2.57, N 10.81%. Found: C 43.52, H 2.50, N 10.88%. IR (KBr, cm^‒1^): 3067 (w), 2938 (w), 1724 (s), 1676 (s), 1590 (s), 1433 (m), 1347 (s), 1294 (s), 1247 (s), 1028 (m), 871 (m), 833 (m), 762 (s), 632 (m), 528 (s).

### 2.3. Synthesis of [Cd(SCN)_2_(DPNDI)] *(**2**)*

A solution of Cd(SCN)_2_ (45.6 mg, 0.2 mmol) in CH_3_OH (5 mL) was carefully layered onto a solution of DPNDI (21.0 mg, 0.05 mmol) in *N,N′*-dimethylacetamide (5 mL) in a test tube. The solution was left to stand for several days in the dark at room temperature, and yellow crystals of **2** were obtained (yield: 58% based on DPNDI). Anal. Calcd for C_26_H_12_CdN_6_O_4_S_2_: C 48.07, H 1.85, N 12.94%. Found: C 48.73, H 1.98, N 13.06%. IR (KBr pellet, cm^−1^): 3596 (w), 3510 (w), 3057 (w), 2095 (s), 1714 (m), 1667 (s), 1576 (s), 1442 (m), 1342 (m), 1243 (s), 1185 (s), 976 (m), 833 (m), 751 (s), 632 (m), 523 (s).

### 2.4. Synthesis of [Cd(DPNDI)_2_(DMA)_2_]·2ClO_4_ *(**3**)*

A solution of Cd(ClO_4_)_2_·6H_2_O (83.9 mg, 0.2 mmol) in CH_3_OH (5 mL) was carefully layered onto a solution of DPNDI (21.0 mg, 0.05 mmol) in *N,N′*-dimethylacetamide (5 mL) in a test tube. The solution was left to stand for several days in the dark at room temperature, and light-yellow crystals of **3** were obtained (yield: 45% based on DPNDI). Anal. Calcd. for C_56_H_42_CdCl_2_N_10_O_18_: C 50.67, H 3.17, N 10.55%. Found: C 50.93, H 3.41, N 11.08%. IR (KBr, cm^‒1^): 3229 (br, w), 2938 (w), 1714 (m), 1676 (m), 1609 (s), 1499 (m), 1400 (m), 1342 (s), 1254 (s), 1080 (s), 971 (s), 890 (s), 789 (s), 590 (m), 513 (m). 

### 2.5. X-ray Data Collection and Structure Refinement

The crystallographic data for compounds **1**, **2**, and **3** were collected at 153 K using a Rigaku-AFC7 (Tokyo, Japan) equipped with a Saturn CCD area-detector system. Measurements were made by using graphite-monochromatic Mo Kα radiation (λ = 0.71073 Å). The frame data were integrated, and absorption correction was calculated using the Rigaku CrystalClear program package (Tokyo, Japan). All the structures were solved by direct methods with the SHELXS-97 program (Göttingen, Germany) [36], and refined by full-matrix least-squares methods on F^2^ using the SHELXL-2014 program package (Göttingen, Germany) [37]. All non-hydrogen atoms were refined anisotropically, and hydrogen atoms of aromatic rings were placed at calculated positions and refined isotropically using a riding mode. The crystal data and the structure refinements are summarized in Table 1. Crystallographic data have been deposited in the Cambridge Crystallographic Data Center as supplementary publication number CCDC 1,812,274–1,812,276 for **1**–**3**, which can be obtained free of charge from the Cambridge Crystallographic Data Centre via www.ccdc.cam.ac.uk/data_request/cif. Experimental details for X-ray data collection and the refinements are summarized in Table 1.

## 3. Results and Discussion

### 3.1. Crystal Structure

**Figure 1:**
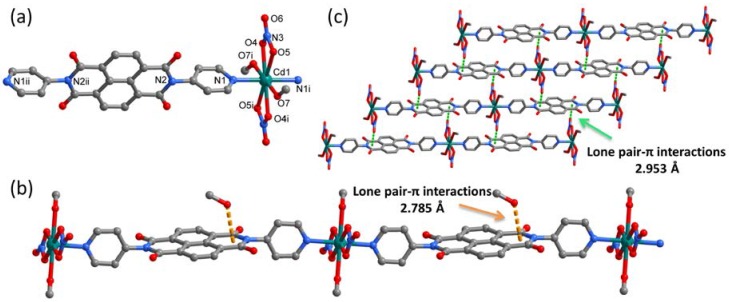
(**a**) Coordination environment of Cd^2+^ in **1**, symmetry codes: (i) 2 – x, –y, 1 – z; (ii) –x, 2 – y, –z; (**b**) the lone pair-π interactions between NDI units and methanol; and (**c**) the lone pair-π interactions extending the one-dimensional chains into a two-dimensional supramolecular network. All hydrogen atoms are omitted for clarity.

Single-crystal X-ray analysis reveals that compound **1** is a 1D coordination polymer. There are half of a Cd^2+^ ion, one half of a DPNDI, one NO_3_^−^ anion, one coordinated methanol molecule, and one free methanol molecule in the asymmetric unit. As shown in Figure 1a, each Cd cation is coordinated in hexagonal bipyramidal geometry by four oxygen atoms from two NO_3_^−^ anions, two oxygen atoms from two methanol molecules (*d*_Cd-O_ = 2.160–2.517 Å) and two nitrogen atoms from two electron-deficient DPNDI ligands (*d*_Cd-N_ = 2.295 Å). The neighboring two Cd cations are bridged by DPNDI ligands to form a one-dimensional coordination polymer (Figure 1b). For each DPNDI, the dihedral angle between NDI core and pyridine group is around 83.9°, which is slightly larger than that of free ligand [38].

In the one-dimensional linear networks, each smallest repetitive unit contains one free methanol molecule which is pointed to the imide ring of DPNDI unit through lone pair-π interaction. The distance between the oxygen lone-pair electrons in methanol molecule and the imide ring is only 2.785 Å (Figure 1b), which is much shorter than that reported previously [38]. Interestingly, the neighboring two one-dimensional coordination polymers are connected each other through another lone pair-π interactions between the lone pair-bearing coordinated NO_3_^−^ anions and imide ring of DPNDI, to generate a two-dimensional supramolecular network (Figure 1c). The distance between oxygen atoms of coordinated NO_3_^−^ anions and the imide rings is 2.953 Å. These 2D networks are further connected by van der Waals interactions to form a three-dimensional supramolecular architecture.

**Figure 2:**
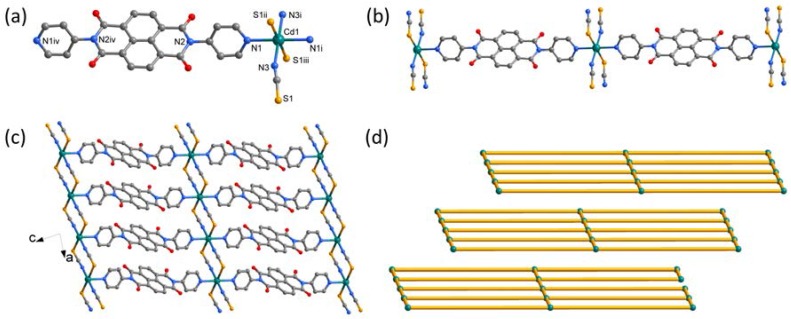
(**a**) The coordination environment of the Cd atom in **2**, symmetry codes: (i) 1 – x, –y, –z; (ii) –1 + x, y, z; (iii) 2 – x, –y, –z; (iv) –x, 1 – y, 1 – z; (**b**) the one-dimensional coordination configuration; (**c**) the two-dimensional framework view along the *ac* plane; and (**d**) the topological structure of **2**. All hydrogen atoms are omitted for clarity.

Single-crystal X-ray diffraction analysis reveals that **2** crystallizes in the triclinic space group $P\bar{1}$ and is composed of a two-dimensional framework. The asymmetric unit of **2** contains a half of Cd^2+^ cation, a half of DPNDI ligand and one SCN^−^ anion (Figure 2a). The Cd^2+^ cation has a slightly distorted octahedral coordination environment, with the site occupied by two nitrogen atoms from two DPNDI, two nitrogen atoms from two SCN^−^ anions (*d*_Cd-N_ = 2.265–2.381 Å), and two sulfur atoms from two other SCN^−^ anions (*d*_Cd-S_ = 2.709 Å). Each Cd^2+^ cation is connected by two DPNDI ligands to form a one-dimensional coordination configuration (Figure 2b). As shown in Figure 2c, the one-dimensional units are connected each other by S and N atoms of SCN^−^ anions resulting in a two-dimensional grid network with the Cd···Cd separations of 5.771 Å. The metal ions play a role as 4-connecting nodes in this grid network. If the DPNDI node is represented by the N–N bond, two thiocyanates node is represented by N–S bond, connections between the metal nodes, the DPNDI and thiocyanates nodes alternately will bring about a 2D (4, 4) topological network, as shown in Figure 2d; and there are π···π stacking interactions (3.358 Å) between the naphthalene rings in the (4, 4) grid. In the crystal lattice, such 2D networks are piled up in parallel and separated by solvent molecules.

**Figure 3:**
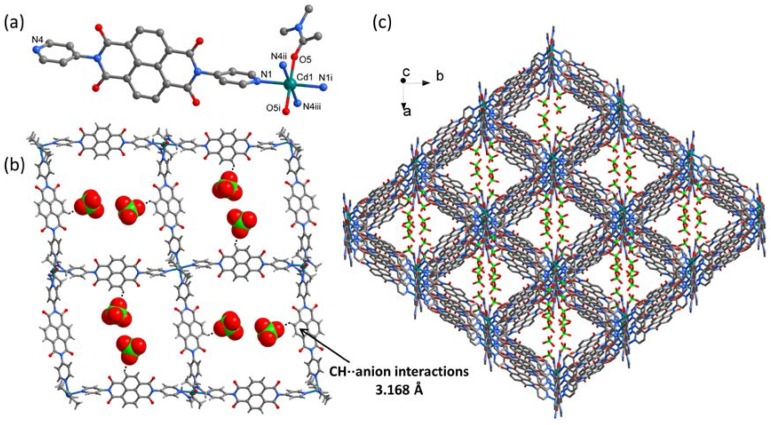
(**a**) The coordination environment of the Cd atom in **3**, symmetry codes: (i) 1 – x, –y, 2 – z; (ii) 0.5 + x, 0.5 – y, –0.5 + z; (iii) 0.5 – x, –0.5 + y, 2.5 – z; (**b**) the square-grid framework, in which ClO_4_^–^ anions were filled in the cavities through CH···anion interactions; and (**c**) the packing diagram for **3** viewing along the c axis.

Single-crystal X-ray diffraction analysis reveals that **3** crystallizes in the monoclinic space group *C*2/*c* and is composed of an extrodinarily large, cationic square-grid framework carrying two ClO_4_^−^ anions in each square cavity. The asymmetric unit consists of one half of Cd^2+^ cation, one DPNDI ligand, one DMA molecule and one ClO_4_^−^ anion (Figure 3a). It should be pointed out that the diffraction data were treated by the SQUEEZE during the refinement to remove diffuse electron density associated with these largely disordered solvent molecules [39]. Each octahedral Cd^2+^ cation is linked by four equatorial DPNDI tectons through the formation of Cd–N bonds (*d*_Cd–N_ = 2.352 Å) and capped by two O atoms from two DMA molecules in axial directions through the formation of Cd–O bonds (*d*_Cd–N_ = 2.301 Å), which leads to a non-interpenetrated and cationic two-dimensional network with square cavities of around 20 Å × 20 Å (Figure 3b). In a given grid, the Cd^2+^ cations are responsible for the square-grid architecture, the ClO_4_^−^ anions play important roles in preventing interpenetration of networks by partially occupying the cavities and aligning planar network sheets parallel to each other by participating in CH···anion interactions with core-Hs of DPNDI (*d*_C–O_ = 3.168 Å) (Figure 3b). Along *c* axis, the neighboring two-dimensional networks are further bridged by ClO_4_^−^ anions through another CH···anion interactions to form a three-dimensional framework with one-dimensional channels filled by ClO_4_^−^ anions and solvent molecules (Figure 3c).

### 3.2. Photochromic Properties

**Figure 4:**
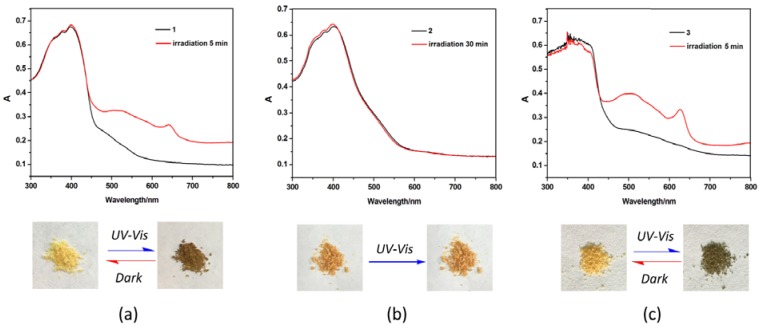
UV−Vis diffuse reflectance spectra of **1**–**3** before and after irradiation (**upper, 1** (**a**)**, 2** (**b**)**, 3** (**c**)); photographs showing the photochromic behavior of **1**–**3** (**bottom**).

Compounds **1** and **3** are photosensitive, giving a color change from yellow into brown or dark brown upon exposure to UV–VIS light in air within 5 min (Figure 4). However, no color changes are observed for compound **2** even when irradiated for 30 min. The photoproducts of **1** and **3** are stable in air, but can return to yellow in a dark room for one day at room temperature. In addition, such decolored samples can also display color changes after irradiation again, which indicates reversible photochromism of **1** and **3**. X-Ray crystallography and PXRD reveals that the crystal structures of photoproducts of **1** and **3** are identical to original of **1** and **3** (Figures S4–S6), but their UV–Vis spectra are different (Figure 4). This phenomenon indicates that the photo-responsive behaviors may be a result from an electron-transfer chemical process in the structure, and not from a structural transformation. As shown in Figure 4, the original of **1**–**3** show strong absorption bands below 500 nm, which corresponds to the n–π* and π–π* transition of the conjugated aromatic ligand [40]. After irradiation, the photochromic products of **1** and **3** show new characteristic bands around 520 nm and 645 nm. These characteristic spectral bands are similar to that observed for NDI radical, suggesting that the color changes of **1** and **3** may arise from the photo-induced generation of radicals in DPNDI molecules [29,30,41].

**Figure 5:**
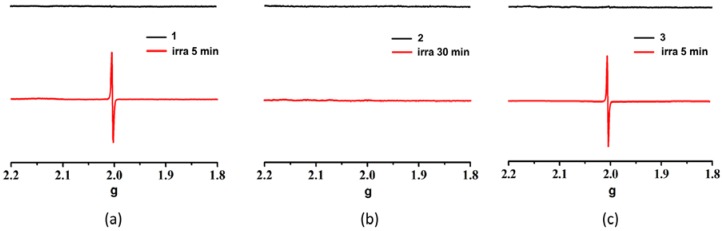
ESR spectra of compounds **1** (**a**), **2** (**b**), and **3** (**c**) before and after irradiation.

To confirm the generation of the radicals, the electron spin resonance (ESR) spectra of **1**–**3** were measured before and after irradiation (Figure 5). Compounds **1** and **3** exhibit no ESR signal before irradiation, but strong ESR signals at 2.0026 and 2.0019 for compounds **1** and **3** are observed after irradiation, which are similar to those found in NDI-based coordination polymers [29,30,32,40]. This indicates that DPNDI ligand is indeed reduced to generate DPNDI^−^ free radicals after two compounds are irradiated. On the contrary, **2** shows no ESR signals before and after irradiation (Figure 5c), which evidences, again, the silence of its photochromism.

### 3.3. Luminescence

**Figure 6:**
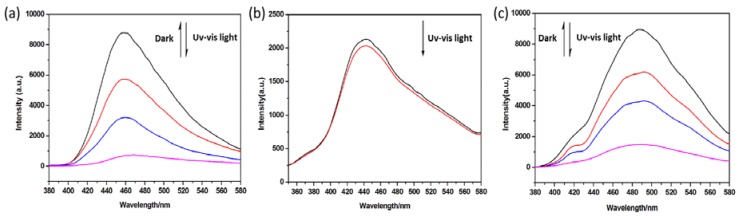
The photocontrolled tunable luminescence performance of **1** (**a**), **2** (**b**), and **3** (**c**), the spectra of **1** and **3** are measured every 2 min upon irradiations (300 W xenon lamp). The spectra of **1** and **2** were recorded upon irradiations (300 W xenon lamp) at every 2 minutes, the spectra of **2** were recorded before radiation (black line) and after radiation for 30 min (red line).

Coordination polymers containing metal centers with the d^10^ electron configuration have been attracting more interest because of their wide-spread applications in chemical sensors, labeling, and photo-chemistry [42]. Considering the luminescence performance of DPNDI (Figure S7), the d^10^ Cd(II) metal ion, and the photochromism of **1** and **3**, the photo-controlled luminescence of these compounds were studied in the solid state at room temperature. As shown in Figure 6, compound **1**-**3** can exhibit blue luminescence with emission maximum at 462, 448, and 495 nm, respectively, upon excitation at 350 nm. For compounds **1** and **3**, photocontrolled tunable luminescences are observed under the trigger of ultraviolet visible light. The strength of the emission of **1** and **3** are gradually reduced under ultraviolet visible light irradiation, and the luminescence is almost quenching when the samples change to dark brown after irradiating for six minutes (Figure 4a,c), but their luminescence can be recovered when the samples stand in the dark for several hours. These distinct behaviors indicate that the photocontrolled tunable luminescence of **1** and **3** are reversible, which further demonstrate that there are electron-transfer interactions among the components of the compounds during irradiation, thus resulting in weaker luminescence and even quenching. However, for compound **2**, almost no obvious changes are observed even upon irradiation for 30 min (Figure 4b).

On the basis of the above results, the anions should play a very important role in determining the structures of the three Cd(II) cadmium coordination polymers. The nature (coordinating ability and geometry) of the anions is the primary reason. In **1**, the NO_3_^−^ anion coordinates to the Cd(II) cation in a terminal chelating mode, which prevents the structure extending into a higher dimension. When the anions are changed to SCN^−^, the 2D layered networks of **2** are obtained, due to the adjacent 1D chains bridged by SCN^−^. Compared with NO_3_^−^ and SCN^−^, the coordinating ability of ClO_4_^−^ is worst in the construction of coordination polymers. The ClO_4_^−^ cannot coordinate to the Cd(II) cation in the self-assembly of **3** and it is located at the 1D channel as a counter ion. It is known that MeOH and DMA are electron-donating species, which can give electron in redox photochromic metal complexes [43,44]. Therefore, the radical generation in **1** and **3** are related to electron transfer pathway from solvent molecules (based on TGA data, Figures S1–S3), but not the anions to DPNDI, which coincides with the recent examples [44,45]. For **2**, because S atoms of SCN^¯^ anions coordinated to the Cd(II) cation, they can remotely donate some electrons from the electronegative S atoms to the electron-deficient NDI cores, which will slightly decrease the π-acidities of the DPNDI (increase the LUMO level of DPNDI) [46]. In other words, the electron-accepting ability of the DPNDI in **2** is slightly decreased by the coordinated SCN^−^ anion, which probably results in the energy level not matching between the solvent molecules and electron-deficient DPNDI electron-deficient NDI. Thus, only **1** and **3** can undergo a photoinduced radical generation upon irradiation, while **2** is silent to the light.

## 4. Conclusions

In summary, three coordination polymers were successfully constructed from the DPNDI ligand and cadmium ions with different counter anions. Compound **1** displays the one-dimensional linear network, **2** displays the two-dimensional network with the (4, 4) net topology, and **3** is a non-interpenetrated square-grid coordination polymer containing one-dimensional rhomboid channels. The structural differences between compounds **1**, **2**, and **3** reveals that the coordination abilities and geometries of counter anions have a significant influence on the assembly procedure. Moreover, due to the different electron donating abilities of counter anions, they exhibit different photochromic behaviors upon irradiation. These studies demonstrate that counter anions not only affect the geometry of coordination polymers, but also can effectively control the photochromic properties of coordination polymers.

## Figures and Tables

**Table 1:** Crystallographic data and structure refinement details for **1**−**3**.

Compound	1	2	3
Chemical formula	C_28_H_20_CdN_6_O_14_	C_26_H_12_CdN_6_O_4_S_2_	C_56_H_42_CdCl_2_N_10_O_18_
formula weight	776.91	648.94	1326.29
crystal system	triclinic	triclinic	monoclinic
space group	$P\bar{1}$	$P\bar{1}$	*C*2/*c*
a (Å)	7.3845 (4)	5.7707 (4)	18.821 (4)
b (Å)	9.2831 (4)	10.7235 (11)	27.177 (5)
c (Å)	12.4333 (6)	16.9651 (16)	17.704 (4)
α (deg)	73.934 (4)	87.406 (8)	90
β (deg)	87.906 (4)	82.664 (7)	108.97 (3)
γ (deg)	68.292 (5)	81.112 (7)	90
V (Å3)	758.87 (7)	1028.39 (16)	8564 (3)
Z	1	1	4
ρcalc(g/cm^3^)	1.700	1.048	1.029
μ (Mo Kα)·(mm^−1^)	0.802	0.661	0.373
F(000)	390	322	2696
collected reflns	5405	7524	29874
unique reflns/ *R*_int_	2492/0.0183	3509/0.0746	7543/0.070
no. of observations	2433	2641	6625
GOF	1.024	1.033	1.160
*R*_1_^a^, *wR*_2_^b^ (*I* > 2σ(*I*))	0.0315, 0.0907	0.0824, 0.2206	0.0816, 0.1821
*R*_1_^a^, *wR*_2_^b^ (all data)	0.0322, 0.0912	0.0982, 0.2402	0.0921, 0.1882

^a^*R*_1_ = Σ||F_o_| − |F_c_||/Σ|F_o_|. ^b^ *wR*_2_ = [Σw(F_o_^2^ − F_c_^2^)^2^/Σw(F_o_^2^)]^1/2^.

## Supplementary Materials

Luminescence spectra of ligand, TG curves of compounds **1**–**3**, and PXRD. The supplementary materials are available online at http://www.mdpi.com/2073-4360/10/2/165/s1.
