# Supplementary material for: Anion-Controlled Architecture and Photochromism of Naphthalene Diimide-Based Coordination Polymers
Source: Polymers (Basel). 2018 Feb 8;10(2):165. doi: 10.3390/polym10020165 (PMC6415121; doi:10.3390/polym10020165)
Supplement: Supplementary file 1 [file polymers-10-00165-s001.pdf]

Electronic Supplementary Information for

Anion-controlled architecture and photochromism of naphthalene diimide-based coordination polymers

Jian-Jun Liu [1,*], Shu-Biao Xia [1], Yu-Lian Duan [1], Teng Liu [1], Fei-Xiang Cheng [1], Cheng-Ke Sun [1]

1 Center for Yunnan-Guizhou Plateau Chemical Functional Materials and Pollution Control, Qujing Normal University, Qujing 655011, China

E-mail: jianjun_liu@mail.qjnu.edu.cn

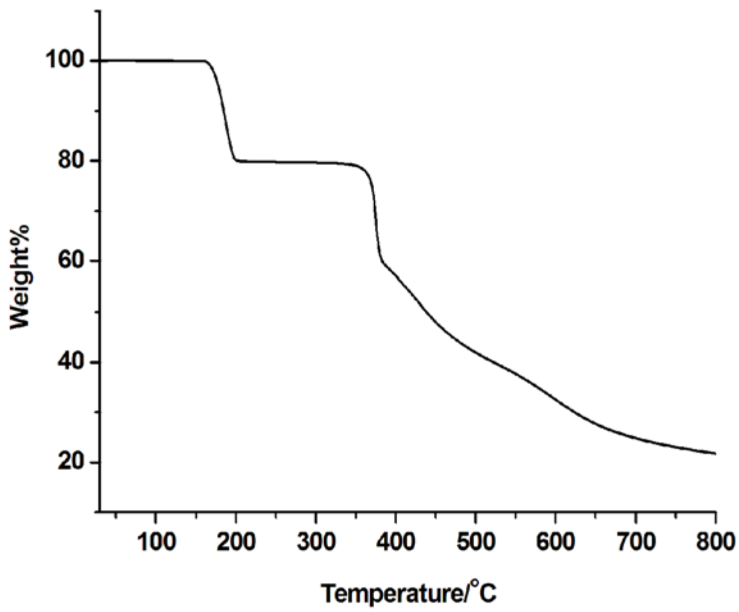

Figure S1. Thermogravimetric (TG) trace of **1**.

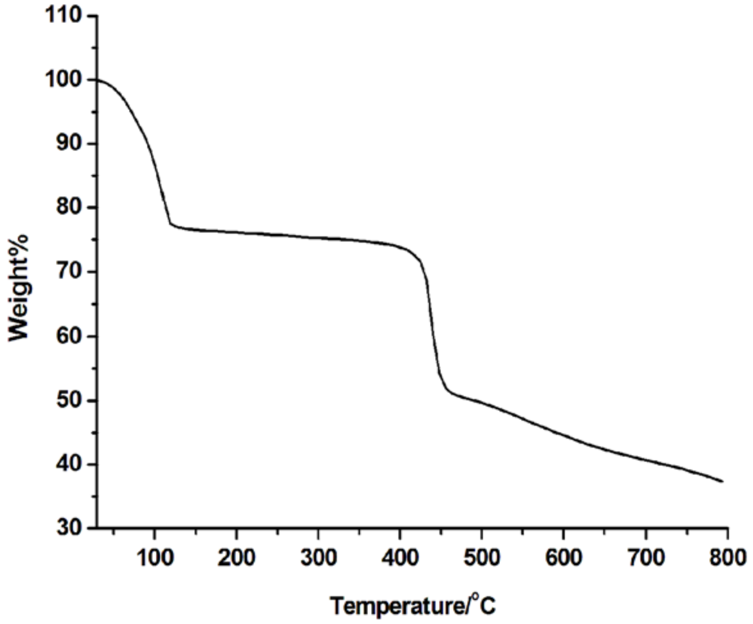

Figure S2. Thermogravimetric (TG) trace of **2**.

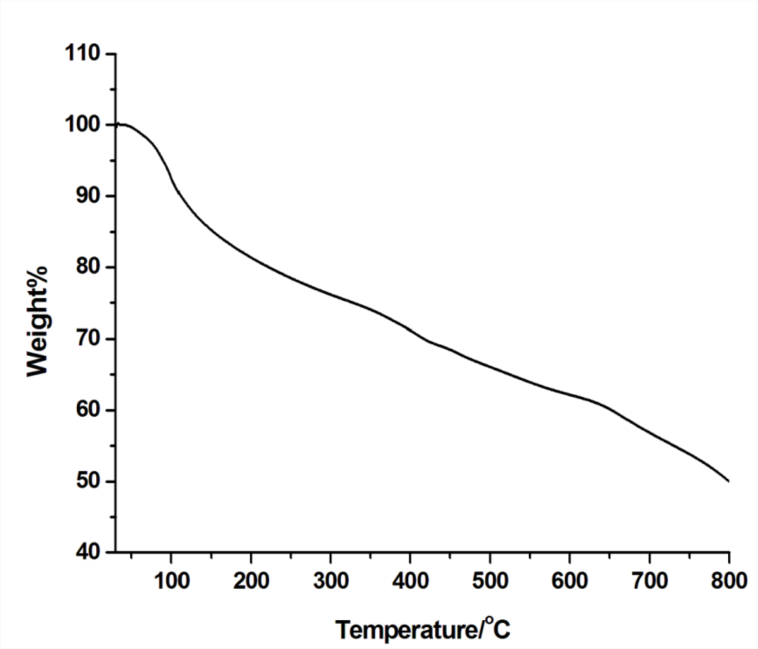

Figure S3. Thermogravimetric (TG) trace of **3**.

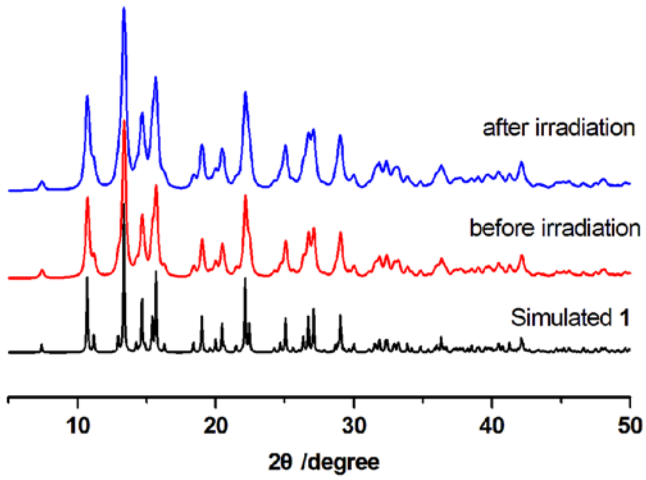

Figure S4. The PXRD patterns for compound 1.

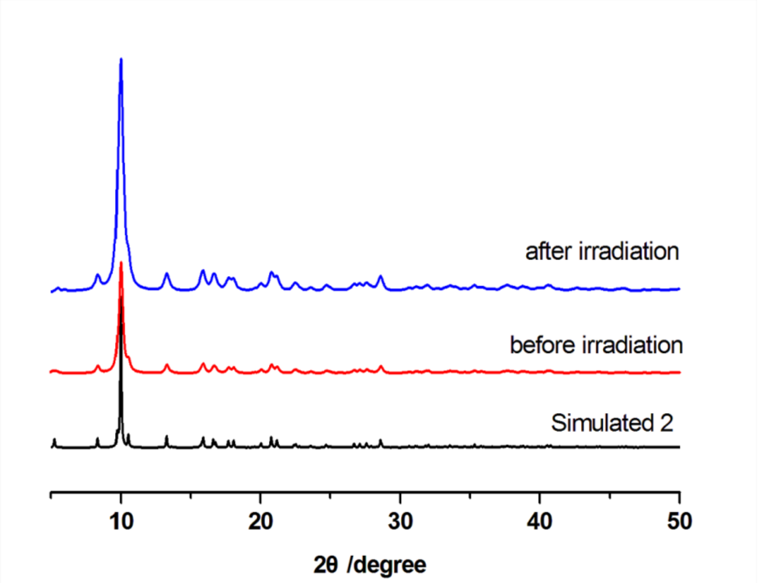

Figure S5. The PXRD patterns for compound **2**.

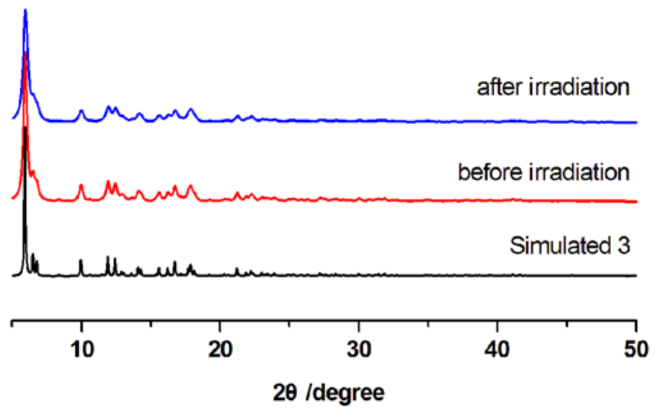

Figure S6. The PXRD patterns for compound **3**.

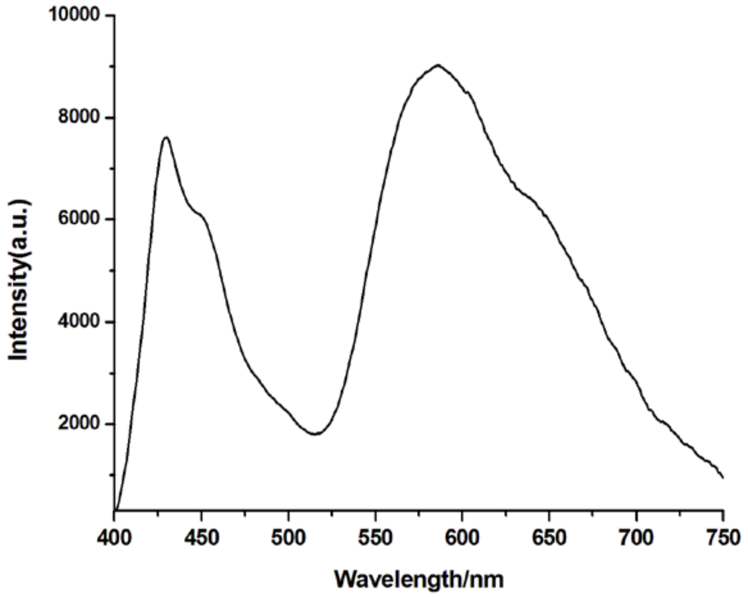

Figure S7. The emission spectra of DPNDI in solid state (excited at 350 nm).
